# Using HPV vaccination for promotion of an adolescent package of care: opportunity and perspectives

**DOI:** 10.1186/1471-2458-13-493

**Published:** 2013-05-21

**Authors:** Catherine MacPhail, Emilie Venables, Helen Rees, Sinead Delany-Moretlwe

**Affiliations:** 1 Wits Reproductive Health and HIV Institute (WRHI), School of Clinical Medicine, Faculty of Health Sciences, University of the Witwatersrand, Johannesburg, South Africa; 2 Collaborative Research Network in Mental Health and Well-being in Rural and Regional Communities, University of New England, Level 1 Botany Building S2, Armidale, NSW, 2351, Australia

## Abstract

**Background:**

Adolescents are a difficult population to access for preventive health care, particularly in less resourced countries. Evidence from developed countries indicates that the HPV vaccine schedule may be a useful platform from which to deliver other adolescent health care services. We conducted a qualitative cross sectional study to assess the potential for using the HPV vaccine in the South African public health care system as an opportunity for integrated health care services for adolescents.

**Methods:**

Parents, young adolescents, community members and key informants participated in interviews and focus group discussions about feasibility and acceptability, particularly the use of the HPV vaccination as the basis for an integrated adolescent package of care. Health care providers in both provinces participated in focus group discussions and completed a pairwise ranking exercise to compare and prioritise interventions for inclusion in an adolescent package of care.

**Results:**

Participants were in favour of integration and showed preference for detailed information about the HPV vaccine, general health information and specific sexual and reproductive health information. Among health care workers, results differed markedly by location. In North West, prioritisation was given to information, screening and referral for tobacco and alcohol abuse, and screening for hearing and vision. In Gauteng integration with referral for male circumcision, and information, screening and referral for child abuse were ranked most highly.

**Conclusions:**

There is generally support for the delivery of adolescent preventive health services. Despite national priorities to address adolescent health needs, our data suggest that national policies might not always be appropriate for vastly different local situations. While decisions about interventions to include have traditionally been made at country level, our results suggest that local context needs to be taken account of. We suggest low resource strategies for ensuring that national policies are introduced at local level in a manner that addresses local priorities, context and resource availability.

## Background

Almost one-quarter of the world’s population are adolescents, with the majority living in low- and middle-income countries (LMIC) [1]. There is increasing recognition that decisions, behaviours and health interventions adopted by adolescents during this stage of life frequently have consequences for morbidity and mortality that extend well into late adulthood [2]. Since 2006, two vaccines have been licensed for the prevention of cervical cancer. Several clinical trials have demonstrated both the bivalent and the quadrivalent vaccine to be safe and efficacious in preventing over 90% of infections caused by HPV types 16 and 18, the HPV types implicated in causing an estimated 70% of cervical cancers globally [3]. Prophylactic vaccines have the greatest impact when administered prior to disease exposure and thus HPV vaccination targeted at adolescent girls has been successfully integrated into public health services in a range of countries including the United States, United Kingdom and Australia, despite some initial challenges with regard to acceptability [4-7]. Public sector delivery has been slower in less developed countries, largely due to cost and challenges with delivery in under-resourced health systems [8,9].

Adolescents are a challenging population to reach with preventive health care service [10,11]. Experience from high income countries has shown that high levels of vaccine uptake have been achieved through school-based vaccination programmes [12]. Evidence from these countries also suggests that adolescents use scheduled HPV vaccination visits to health care providers (HCP) to address other health care requirements [13] A study of adolescent women using academic health centres and private practices in the US for HPV vaccination noted that almost half used their vaccine visit to receive other medical or preventive services [14]. The World Health Organization (WHO) has recommended leveraging this potential symbiosis by suggesting that the required HPV vaccination schedule be used as a platform from which to deliver screening programmes, provision of information, services, commodity delivery or other vaccines, particularly in LMIC (see Table 1) [15]. In LMIC health care delivery for adolescents is challenging as many services included in maternal and child health programmes do not currently extend beyond early childhood. Advances in HPV prevention through vaccination, as well as the potential for HIV vaccines, are predicted to drive even the most under-developed countries to examine the potential for reaching adolescents and pre-adolescents with vaccine programmes [16]; increasing adolescent access to other health care services. Currently available HPV vaccine delivery mechanisms include expanding the existing state immunization programme or developing a school-based vaccine programme [17].

**Table 1:** **Suggested additions to an adolescent HPV vaccine**-**based package of care** (“**Adolescent Plus Package**”) [[18]]

**Intervention category**	**Definition**	**Menu of complimentary interventions**
•Screening	•Testing or screening for illnesses, diseases or disabilities	•Vision screening
		•Schistosomiasis screening (in appropriate contexts)
		•Anaemia screening
•Provision of information	•Providing information-based interventions, including information sessions, skills delivery, pamphlets	•HPV information
		•Reproductive and sexual health information booster
		•Nutritional information
		•Tobacco and alcohol prevention information
•Services	•Providing referrals, counseling, treatments	•Voucher referral for health services (including reproductive health)
		•Referral for vision
		•Referral for iron/folate supplementation
•Commodity delivery	•Supplementation, direct provision of commodities or increasing access to commodities	•Anti-helminthic (soil-transmitted)
		•Anti-helminthic (schistosomiasis)
		•Vitamin A
		•Bednets for malaria prevention
•Vaccines	•Vaccines identified to complement HPV vaccine delivery should already be delivered (primary or booster) to this age group	•Tetanus/Diptheria booster
		•Hepatitis B
		•Meningococcus
		•Typhoid
		•Rubella

In South Africa, over 51% of the population is now under the age of 25 years. Of those, 41% are between the ages of 10 to 19 years, and 90% are enrolled in school [19,20]. Challenges in reaching adolescents through traditional health care services and high levels of school enrolment suggest that school-based HPV vaccination would potentially be the most effective delivery strategy. Thoughts about the introduction of the HPV vaccination to the South African public sector are timely, given that the South African government has recently re-launched the Integrated School Health Programme (ISHP) based on WHO recommendations for School Health Programmes [21]. South Africa has had a school health programme since 2003 but implementation has been varied in both quality and geographic distribution. Challenges have included lack of staff, resources and referrals as well as a poor environment in which to provide service delivery and lack of access to some schools, particularly in rural areas [22]. The new draft ISHP will be implemented by the Departments of Health, Basic Education and Social Development and will take into account restructuring of primary health care. Specifically the policy seeks to provide preventive and health promoting services, identify and address health barriers to learning, facilitate access to services and to support the school community in creating a safe and secure environment for teaching and learning [21]. The draft policy makes provision for health education, health screening and provision of on-site services; with a particular emphasis on sexual and reproductive health (SRH) for learners in grades 4–12 (see Table 2). The services outlined are a suggested minimum starting point, with new on-site services to be added over time. Currently the ISHP includes immunization in only the Foundation phase, but the recent success of a measles booster vaccination programme in Gauteng schools to combat the 2010 measles outbreak suggests that schools are well placed to act as vaccine sites for the HPV vaccine programme, once initiated.

**Table 2:** **Summary of school health package** [[21]]*

**Health screening**	**On-site service**	**Health education**
**Foundation phase (Gr R-3) [approx. age 5–7 years]**		
•Oral health	•Deworming (includes bilharzia and malaria control where appropriate)	•Hand washing
•Vision	•Immunisation	•Personal & environmental hygiene
•Hearing	•Oral health (where available)	•Nutrition
•Speech	•Minor ailments	•Tuberculosis
•Nutritional assessment		•Road safety
•Physical assessment (Gross & fine motor)		•Poisoning
•Mental Health		•Know your body
•Tuberculosis		•Abuse (sexual, physical and emotional abuse)
•Chronic illnesses		
•Psychosocial Support		
**Intermediate phase (Gr 4–6) [approx. age 9–11 years]**		
•Oral health	•Deworming	•Personal & environmental hygiene
•Vision	•Minor ailments	•Nutrition
•Hearing	•Counselling and referral for sexual and reproductive health as needed	•Tuberculosis
•Speech		•Medical and Traditional Male circumcision
•Nutritional assessment		•Abuse (sexual, physical and emotional abuse including bullying, violence)
•Physical assessment		•Puberty ( e.g. physical and emotional changes, menstruation & teenage pregnancy)
•Mental Health		•Drug & substance abuse
•Tuberculosis		
•Chronic illnesses		
•Psychosocial Support		
**Senior phase (Gr 7–9) [approx. age 12–14 years]**		
•Oral health	•Minor ailments	•Personal & environmental hygiene
•Vision	•Counselling and referral for sexual and reproductive health as needed	•Nutrition
•Hearing		•Tuberculosis
•Speech		•Abuse (sexual, physical and emotional abuse including bullying, violence)
•Nutritional assessment		•Sexual & reproductive health
•Physical assessment		○Menstruation
•incl. anaemia		○Contraception
•Mental Health		○STIs incl. HIV
•Tuberculosis		○MMC & Traditional
•Chronic illnesses		○Teenage pregnancy, CTOP, PMTCT
•Psychosocial support		○HCT & stigma mitigation
		•Drug and substance abuse
		•Suicide
**Further Education and Training (FET) (Gr 10–12) [approx. age 15–17 years]**		
•Oral health	•Minor ailments	•Personal & environmental hygiene
•Vision	•Counselling and referral for sexual and reproductive health as needed	•Nutrition
•Hearing		•Tuberculosis
•Speech		•Abuse (sexual, physical and emotional abuse including bullying, violence)
•Nutritional assessment		•Sexual & reproductive health
•Physical assessment incl. anaemia		○Menstruation
•Mental Health		○Contraception
•Tuberculosis		○STIs incl. HIV
•Chronic illnesses		○MMC & Traditional
•Psychosocial support		○Teenage pregnancy, CTOP, PMTCT
		○HCT & stigma mitigation
		•Drug and substance abuse
		•Suicide
•**All schools**		
	•Environmental assessment	
	•First aid kit	
	•Water and sanitation	
	•Cooking area	
	•Physical safety	
	•School nutrition programmes (where appropriate).	

*STIs* sexually transmitted infections, *MMC* medical male circumcision, *CTOP* choice of termination of pregnancy, *PMTCT* prevention of mother-to-child transmission of HIV, *HCT* HIV counselling and testing.

*Each child should be assessed through health screening at least once in each of the four educational phases; more frequently (on request) in the case of sub-optimal learning. There is currently no detail in the draft plan on frequency of on-site services and health education but it appears that the intention is for this to be on-going.

A recent systematic review of the role of school-based health care in adolescent sexual, reproductive and mental health highlighted the importance of exploring sources of resistance and support in the community to school-based health services [23]. We conducted a study to assess the acceptability and feasibility of introducing HPV vaccination in the South African public health sector, and particularly the notion of using the vaccination schedule of three repeat visits as the basis for providing adolescents with additional health services. In particular, we used a novel participatory exercise to determine priority setting around potential services in two different locations, one rural and one urban, to highlight any potential influences that setting and access to resources might have on choices.

## Methods

A cross-sectional, qualitative observational study was conducted in Johannesburg, Gauteng and Taung, North West Province in South Africa. The two sites represent the two extremes of development in South Africa; Johannesburg being the major economic urban centre of the country and Taung, a small rural town with limited development and infrastructure. Access to health care in both the private and public sector is limited in Taung in comparison to Johannesburg.

### Sampling

Key informants working provincially and nationally in cancer prevention, immunisation, sexual health, adolescent health and education were contacted for interviews. Referrals were made at each interview for other potential participants. In each province a single region or sub-district was selected and within each, a health care facility and schools were asked to participate. Selection of health care facilities and schools ensured representation of the socio-demographics of the country, based on socio-economic status of users. In each province data collection was conducted with adolescents, parents, community members, teachers and health care providers using qualitative methods including In-depth Interviews (IDIs) and Focus Group Discussions (FGDs). Within health care facilities, HCPs were purposively selected from those involved in the Expanded Immunisation Programme, cervical cancer prevention, SRH services or clinic managers. At schools learners in grades 4–9, aged 9–14 years and their parents were invited to participate in the research. Consent letters informing parents about the data collection were distributed through the schools to be completed by parents and returned to teachers. Community members were recruited from community organisations or through snowballing sampling with known individuals in the communities. In Gauteng, members of an existing local Community Advisory Board that assists the research organization with accessing participants, feedback on proposed protocols and keeping communities informed of study results were invited to participate.

### Data collection

Data collection was conducted from December 2009 to February 2011. A team of seven researchers collected the data. Five had post-graduate degrees in health related disciplines and five were native African language speakers. Three researchers conducted the key informant interviews and one of these with an additional four researchers worked in alternating teams of two (moderator and note taker) on the FGDs and conducted IDIs with African language speakers individually. All researchers were involved in the development of the study, were trained in the study instruments and conducted pilot data collection prior to working with the selected sample.

Youth and parents were recruited for either an FGD or an IDI; participants did not take part in both. Educators and community members participated in FGDs. The number of participants in FGDs ranged from 6 to 13, with a mean of 7 participants. Key informants participated in one-on-one interviews, sometimes telephonically, depending on their location and schedules. All participants were asked to comment on the feasibility and acceptability of a HPV vaccine programme for adolescents, and to specifically comment on integration, using an open-ended semi-structured interview guide.

IDI and FGD topic guides were generally similar and focused on leading participants through thinking about cervical cancer prevention and adolescent health priorities and services, before focusing specifically on the HPV vaccine (acceptability and integration issues). With regard to integration, participants were asked to comment on “What else should be done for adolescents at the time of their HPV vaccination?” Study participants (other than HCPs and key informants) had limited knowledge about the HPV vaccine, and were given minimal information in order to enable them to participate in the study. They were informed that the HPV vaccine prevents cervical cancer in women, and can prevent other cancers in men. Participants were also told that the vaccine was available in the private sector in South Africa, but not in public health facilities. Detailed information was provided at the end of the interview or FGD and all participants were given the opportunity to ask questions. The key informants were also specifically asked to comment on feasibility of adding HPV vaccination to the existing free vaccination programme.

Health care providers in both provinces all participated in two distinct FGDs: one assessing general acceptability and feasibility of introducing the HPV vaccine into the South African public health sector (as above) and the other specifically examining the elements of an adolescent package of care that might be developed alongside the HPV vaccination programme. Health care providers were specifically targeted for this exercise as it was felt that they would have a grasp of potential health interventions beyond that of parents, adolescents and educators. To collect this data a pairwise ranking exercise was completed in which the health care providers were asked to compare and prioritise a range of interventions for inclusion in an adolescent package of care. Pairwise ranking allows for comparison and prioritising within a small list of options and for making decisions in a consensus-orientated manner [24]. A review of the literature and WHO documentation suggesting an adolescent package of care was used to identify current concerns and priorities with regard to adolescent issues in South Africa. Ten different options were included in the pairwise ranking matrix: these included, screening for *hearing and vision*; information, screening and referral for *tobacco and alcohol abuse*; information, screening and referral for *gender*-*based violence* (*GBV*); information, screening and referral for *child abuse*; information and referral for *sexual and reproductive health*; screening and referral for *anxiety and depression*; referral for *male circumcision*; provision of *condoms and*/*or tampons*; assistance with obtaining *vital documents* (i.e. birth certificates and/or identity documents); and provision of *additional vaccines*. We limited the matrix to ten options to prevent participant fatigue as the ranking process is time intensive, even when limited options are provided. Group participants were provided with a matrix listing the range of options and were asked to systematically compare each option with every other option in the matrix. Thereafter, the number of times an option was selected was tallied and the options ranked. While it is tempting to concentrate on the ranking only, the discussion that occurs while participants go through the process of ranking is equally important. During the ranking exercise a researcher facilitated the spontaneous discussion of preferences for particular services. Data collection was conducted in isiZulu in Johannesburg and SeTswana in Taung, and English for key informant interviews.

### Data analysis

The digital recordings of discussions were translated into English and transcribed in a single step. The interviewer checked the transcripts for completeness and accuracy of meaning. Thereafter, the transcripts were read and re-read by the authors to establish the dominant themes emerging from the study as a whole and the various sub-groups of respondents. A framework analysis approach was used to allocate each line of text to a specific code [25]. Codes were grouped according to theme and reviewed again. Analysis continued as an iterative process through discussion and refining of the major themes emerging from the transcripts by the three authors. During the FGD ranking information for each pair was entered onto the matrix by an individual nominated by the rest of the group. Tallying individual choices and final ranking was conducted by one of the authors (CM) once the group discussion was concluded.

### Ethics

Approval for the research was received from the Wits Human Research Ethics Committee (Ref# M090933) and the Departments of Health and Education in both provinces. Each participant provided written informed consent before the start of data collection, both for participation and recording of discussion. For adolescents, parental consent was required before they provided their own assent for participation. The study meets the RATS guidelines on qualitative research.

## Results

Table 3 describes the range of participants, the interview approach and recruitment method. We have focused specifically here on discussions of integrating HPV vaccination with other adolescent health services; general information on acceptability and feasibility of introducing HPV vaccination into the public sector is presented elsewhere [26]. Relatively common themes emerged from discussions across all the participant groups and from both locations on the potential for integrating HPV vaccination with other services. Integration was not discussed in the general focus group discussions with HCPs, as it was the main focus of the pairwise ranking discussions and will be reported on as such.

**Table 3:** **Summary of data collection**, **by participant type**, **method and sampling strategy**

**Participants**	**n**	**Method**	**Recruitment**
Key Informants	25	IDIs	Purposive, snowball
Health Care providers	27	4 FGDs/PWR	Clinics
Educators	11	2 FGDs	Schools
Parents	35	2 FGDs; 18 IDIs	Schools
Youth	63	4 FGDs; 35 IDIs	Schools
Community	32	2FGDs; 12 IDIs	Purposive, snowball
**Total**	**193**		

*IDI* In-Depth Interview, *FGD* Focus Group Discussion, *PWR* Pairwise Ranking.

In the interviews, young people had difficulty articulating what an integrated package of adolescent care might look like. Many responded that they did not know. There was however an expressed demand for specific information about the vaccine that they might receive, with young people noting that there was a need either for someone to ‘. . . *explain to you why they*’*re giving you that and how it can help and what are the symptoms or things which can hurt you*’ rather than ‘*vaccinate and vaccinate and injecting*.’, or to provide pamphlets and books with this information. Other frequent suggestions included providing information about unspecified health issues, and teaching young people ‘. . . *we must not do older people*’*s things at a young age*’ in reference to counselling adolescents to remain sexually abstinent. When specifically asked whether condom provision would be welcomed, there were mixed reactions.

In discussions with parents, the most common recommendations were to include additional diseases (particularly other cancers) in educational messages, and to focus on adolescent reproductive health. One of the parents interviewed commented that:

*They should be taught about prevention of pregnancies*. . . *Whatever you* [*as parents*] *will be telling them*, *they will choose whatever they have decided so I think that they should be taught about infections*, *different infections*. *They should be taught about STIs and they should be taught about pregnancies*, *they should be taught about HIV*. *Our children have chosen*, *they listen to whatever they want to*; *they do what they want to do so I think that we should always preach*…

This demand was specifically about provision of information, rather than actual services, although one parent commented that ‘. . . *they should not be taught about condoms*, *because they are going to try them out*’. Perspectives such as this were in the minority.

Focus group discussions with community members were divided on how HPV vaccination might be integrated with other services. Provision of HIV information and education was immediately raised as a potential add-on with participants noting that ‘. . . *we have to talk about HIV and AIDS because parents don*’*t talk to their kids*’, but others expressed concerns that this topic was inappropriate for children aged 9–14 years. Community members echoed young people’s demands for proper information about the HPV vaccination, and parent’s expressed desire for adolescent reproductive health information, as illustrated by the following community member comment:

*It is important that a girl child be taught about prevention*. *When they have boyfriends* . . . *that when they have boyfriends they will fall pregnant*. *They need to be taught to go to the clinic and get injections*.

The quote above focuses on the provision of information rather than services; there were few participants in these groups who motivated for additional service provision. Educators identified the potential for integration of screening interventions. They tended to focus on additional interventions that might impact on adolescents’ ability to learn in the classroom setting. Their discussions tended to focus on testing of vision or hearing.

Like other participants, many of the key informants focused on the potential for an HPV vaccination to act as a vehicle for general health and reproductive and sexual health information provision; however they went further and identified a range of potential interventions, including screening, treatment and other vaccines that could be linked to the HPV vaccination programme.

*If the vaccine is being administered at age 14 or 15*, *then I think it*’*s the perfect opportunity to link with the sexual and reproductive health programmes and do that in tandem*. *I think the information will need to be adapted if the vaccine was administered at the earlier age of nine or ten*.

*Yeah I think* . . ., *I think there are four linkages*. *The one is Vitamin A supplementation*, *and then the second is de*-*worming*, *and the third is a booster to the vaccination of tetanus and diphtheria*. *I think the fourth*, *depending on resources*, *would be an eye or hearing test*. *So those are four kind of easy screens*. *I think also that one should give information to the mothers of the girls about secondary prevention* [*of cervical cancer*] *and should link it to screening of mothers*.

HPV vaccination integration with other issues was seen as a beneficial way of including young men, who would be unlikely to receive the vaccine, given current recommendations for resource limited countries.

There were however some key informants who advocated that an HPV vaccination programme should be stand alone. Key informants were concerned about budget implications of large-scale integrated programmes and the state’s ability to deliver such programmes.

*I very much like the idea you*’*ve just mentioned of using it as an opportunity*, *but if that*’*s going to compromise the whole programme because it*’*s going to be too complicated and too difficult to do*, *then obviously that*’*s a consideration*. *Personally I*’*m very disappointed at the way our family planning and contraceptive services work*, *that they don*’*t use the interactions with women as an opportunity to do Pap smears*.

### Ranking outcomes

Results differed markedly by location (see Table 4). In North West, participants prioritised combining HPV vaccination with information, screening and referral for substance abuse, while screening for vision or hearing deficiencies was ranked second. In Gauteng, the preference was for combining HPV vaccination of girls with referral of boys for male circumcision. Information, screening and referral for child abuse was ranked second. There was consensus among participants from both provinces about the potential value of combining HPV vaccination with SRH service provision, and of screening for depression and anxiety; items were ranked 3 and 4 respectively. Assistance with obtaining vital registration documents was ranked higher by participants from North West, the more rural province. Screening and referral for gender-based violence was ranked low by both groups, although higher in North West than Gauteng. As well as differences in the potential services ranked most highly, there were provincial differences in the services that were thought to be least useful. In North West, the provision of additional vaccines was ranked lowest, while in Gauteng, providing screening, information and referral for substance abuse was favoured least for inclusion in an integrated package of adolescent health interventions. In addition, both locations did not appear to favour the provision of commodities like condoms and/or tampons at schools.

**Table 4:** Results of pairwise ranking of potential interventions to include with the HPV vaccination in an adolescent health care package

	**North West ranking**	**Gauteng ranking**
Screening for hearing or vision	2	5
Information, screening & referral for tobacco & alcohol abuse	1	10
Information, screening & referral for GBV	6	9
Information, screening & referral for child abuse	8	2
Information & screening for SRH	3	3
Screening & referral for anxiety & depression	4	4
Referral for circumcision	7	1
Provision of condoms &/or tampons	9	7
Assistance with obtaining vital documents	5	6
Additional vaccines (e.g. Diptheria, tetanus or MMR)	10	8

*GBV* Gender Based Violence, *SRH* Sexual and Reproductive Health, *MMR* measles, mumps and rubella.

#### North West perspectives

In rural North West province, HCPs were most enthusiastic about integrating HPV vaccination with the provision of information, screening and referral for tobacco and alcohol abuse, and screening for hearing and vision. Comments by Taung participants seemed to indicate that substance abuse problems are rife among adolescents, and become a concern even at a young age. A Taung female health care provider noted:

*Because you say that vaccine is going to be introduced for age 9 and 14* … *I also think that for tobacco and alcohol abuse thing to get that information is going to be important for them*.

Like educators, HCPs felt that hearing and vision screening are important in a population of school-going age. A female participant indicated that:

*Most of the children at primary level like most of them are still 9 years*, *kids at primary level*, *so I think if they are screening for hearing or vision then it will help them*, *that they can get the vaccine*.

Screening for hearing and vision through an integrated adolescent health package was also viewed as important in catching problems in children who might otherwise not receive care for such issues.

*Sometimes the parents they don*’*t check their kids for disease or things like*. *They are ignorant*. *So if you introduce these things in the schools and then children are screened for hearing and vision they will be able to come with the vaccine*.

Although among Taung participants the addition of other vaccines was ranked the least important potential programme, participants were still relatively positive about it. A male health care provider mentioned:

*Because they are*… *here in the clinic there are so many babies who miss their vaccines* … *so it*’*s better to have additional vaccines*.

The provision of condoms was also ranked as relatively unimportant for adolescents. Although all participants contributed to determining the ranking, the discussion about condoms was dominated by a single male participant who doubted that condom promotion would assist with reductions in HIV. He noted:

*We have to teach our community*. *They must have a vision*, *we are having a problem*…*for example we are having a problem of HIV*, *you must stop having many partners*, *you must have only one partner so if you give them information they must use condoms*, *which means at the end of the day we are not going to fight or win the HIV*, *because if you use a condom it*’*s not 100 per cent safe*.

#### Gauteng perspectives

In contrast, Gauteng HCPs ranked HPV vaccination integration with referral for male circumcision, and information, screening and referral for child abuse most highly. Although Gauteng HCPs ranked circumcision as their most desirable intervention to include with a potential adolescent HPV vaccine programme, the choice was not made easily. There was much debate about the validity of recent clinical trial data supporting the role of circumcision in HIV prevention; however, there was also acknowledgement of the protective role of circumcision with regard to STIs. One of the participants expanded on this when he stated:

*You still owe the guys that are circumcised that are in the risk of infecting these girls*, *circumcised or not circumcised*, *it*’*s the same if they practice unsafe sex*. *If they got more than one partner*, *they are dangerous*. *It is not about the trachoma* [*sic*], *is about the semen that comes from men*. *If you sleep here today and tomorrow there*, *you are spreading something*.

Among Gauteng HCPs screening for child abuse was viewed as an important component of an adolescent intervention given the high rates of child abuse in the communities that they serve.

Gauteng HCPs were least supportive of HPV vaccine integration with alcohol and tobacco screening and referral, and programmes to deal with gender-based violence. As was the case with Taung, lower ranking options were still viewed positively by participants who acknowledged the need for such services. With regard to screening and referral for gender-based violence, a health care provider admitted that this is a serious issue in the Johannesburg inner city:

*Yeah*, *it does happen because I was working in* [. . . ] *Trauma unit*, *and girls that are been abused*, *most of the time they don*’*t talk*, *because they are threatened with death*, *so they should rather die with that*. *Though you could see that* [*there was*] *continuous sexual abuse she will say absolutely nothing*. *I think they need to be given up the information and be referred to the relevant resources*.

## Discussion

Administering the HPV vaccination at schools provides an opportunity to link this activity to an integrated package of care for adolescents in South Africa. Our results indicate that there is generally a positive attitude towards using opportunities presented by HPV vaccine provision for additional adolescent care and services. School-based healthcare is more common in the US and UK than in LMIC, including South Africa [23], however researchers have suggested that such service provision addresses the access needs of young people and specifically appeals to those sub-groups of adolescents who might need them most [27].

In this study, across young people, parents, community members and key informants there was agreement on priorities for adolescent health care. Specifically, adequate information about the HPV vaccine, general health information and SRH information provision were identified; echoing findings from similar studies in other LMIC [28]. Interventions to improve educational outcomes were also prioritised in the form of screening for vision and hearing impairment. The suggestions from participants in this study anticipated many of the components suggested for future implementation in the ISHP, but not all potential components were favoured. In particular, there was little mention of sexual and reproductive health *services*, as opposed to *information*, among participants, other than key informants. The low preference for distribution of commodities like condoms and tampons in the ranking exercise may reflect conservative attitudes of HCPs with respect to adolescent sexuality. Other recent studies of HCPs in South Africa have identified that many believe young women should not have sex before marriage and thought that young women ignore information they receive about HIV and pregnancy prevention. In that study, some services have also been reported to only be available for those over 18 years of age [29]. While this focus on information in preference to services might be interpreted as conservatism about adolescent sexuality, the data do not necessarily support this. Throughout discussions, participants indicated a general willingness and interest in having SRH issues addressed. Alternative interpretations are that; first, the preference for information rather than services at schools reflects the experience and position of respondents; the majority are more familiar with schools as venues for provision of education and information, rather than locations for health services. Second, participants might have perceived information provision as being more feasible than services in school environments. Finally, this may reflect greater comfort with adolescents as learners and recipients of information, rather than active participants in their own health.

The participatory pairwise ranking exercise revealed distinct differences in priority setting between HCPs in the two different locales. While there was positive sentiment about the provision of all suggested services for adolescents, as illustrated through the discussion that accompanied the ranking exercise, the different ranking of priorities between the two groups of HCPs is potentially indicative of different epidemiological contexts or health service access issues in the two provinces. In rural North West, priorities tended to reflect the challenge with accessing the more basic services, such as screening for hearing and vision, which was viewed as beneficial as this service is not routinely being provided. By comparison, in Gauteng where there are greater resources and potentially greater access to preventive health information, the focus was on newer and more innovative prevention strategies for HIV and child abuse prevention. Given that funding for the ISHP is unlikely to be immediately available for the full spectrum of services, research such as presented here may assist in expanding the programme in a manner that specifically addresses perceived needs and service delivery gaps in individual provinces.

To date, there has been a limited but growing literature on the value of packaging health interventions at opportune moments. Kim et al. have applied methods of operations research and cost-effectiveness analysis to inform the optimal selection of health interventions to package together with other single contact interventions such as cervical screening [30]. As part of this work, they have developed an analytic framework, including a binary integer programming model to evaluate candidate packaged interventions, taking into explicit consideration, not only the costs and benefits of interventions, but also the availability of appropriately skilled health care workers to deliver the interventions. While these approaches are useful, they require data on the burden of disease in the target population. Currently the availability of routine data on the burden of disease in 9–14 year old youth in South Africa is limited, although existing survey data indicate that children in this age-group are not accessing or receiving health care services and have a significant burden of untreated illness. By comparison, the ranking exercise, which is a simple participatory exercise that involves HCPs and their knowledge of health care priorities within their context, may be a useful first step in the prioritisation process in settings where routine data are not available, or while more complex models are being developed.

While the data presented here are qualitative, the strength in this study is the ability to compare responses across groups and locations. By qualitative data collection standards, the sample is large, and the responses provided were consistent across groups and locations. We believe that the inclusion of young people’s opinions in this study is a strength, and recognises their importance in the acceptance and uptake of future integrated adolescent health care services at schools.

## Conclusion

Given that South African policy makers are in the process of developing integration strategies for adolescent health priorities, it seems plausible that the HPV vaccine might be assimilated into such decisions and used as a vehicle for driving timing and scheduling of interventions. Decisions about interventions to be included in an ISHP have already been formulated, but our results suggest that an adolescent package of care (with or without the addition of the HPV vaccine) needs to be designed to take account of local context. Limited resources mean that implementation of the ISHP may need to be phased; we suggest that when making decisions about priorities for inclusion in the programme a range of potential interventions is provided and that choices are made at provincial level with reference to context and priorities. Further expansion of services can be achieved in the same manner. While linking interventions to the delivery of the HPV vaccine may have initial additional costs in the short-term, this linkage might well improve effectiveness by addressing the broad range of health issues among adolescents, who are traditionally difficult to reach.

## Abbreviations

CTOP: Choice of Termination of Pregnancy; FGD: Focus Group Discussion; GBV: Gender-Based Violence; HCP: Health Care Provider; HCT: HIV Counselling and Testing; HPV: Human Papilloma Virus; IDI: In-depth Interview; ISHP: Integrated Schools Health Programme; LMIC: Low- and Middle-Income Countries; MMC: Medical Male Circumcision; MMR: Measles, Mumps, Rubella; PMTCT: Prevention of Mother-to-Child Transmission of HIV; PWR: Pairwise Ranking; SRH: Sexual and Reproductive Health; STI: Sexually Transmitted Infections; WHO: World Health Organization

## Competing interests

The authors declare that they have no competing interests.

## Authors’ contributions

CM provided research support during data collection, coded and analysed the data and wrote the paper. EV provided project management, conducted interviews, coded and analysed the data and contributed to the manuscript. HR provided insights on the original design and data collection tools. SD-M conceptualised the original study, reviewed transcripts and made substantial contributions to the manuscript. All authors read and approved the final manuscript.

## Pre-publication history

The pre-publication history for this paper can be accessed here:

http://www.biomedcentral.com/1471-2458/13/493/prepub
